# Association Between Plant‐Based Dietary Indices and Mediterranean Diet Score With the Odds of Gestational Diabetes Mellitus in Iranian Women: A Case–Control Study

**DOI:** 10.1002/edm2.70279

**Published:** 2026-07-11

**Authors:** Mina Bahrami, Farnush Bakhshimoghaddam, Masoume Gharang, Masoud Veissi, Anahita Mansoori

**Affiliations:** ^1^ Student Research Committee Ahvaz Jundishapur University of Medical Sciences Ahvaz Iran; ^2^ Nutrition and Metabolic Diseases Research Center, Clinical Sciences Research Institute Ahvaz Jundishapur University of Medical Sciences Ahvaz Iran; ^3^ Department of Nutrition, School of Allied Medical Sciences Ahvaz Jundishapur University of Medical Sciences Ahvaz Iran

**Keywords:** gestational diabetes mellitus, maternal nutrition, Mediterranean diet score, plant‐based dietary indices, pregnancy

## Abstract

**Introduction:**

Gestational diabetes mellitus (GDM) is a significant pregnancy complication. This study aimed to assess the association between plant‐based dietary indices (PDI) and the Mediterranean Diet Score (MDS) with the odds of GDM in Iranian women.

**Methods:**

A case–control study was conducted involving 276 pregnant women (138 with GDM and 138 controls). Dietary intake was assessed using a validated food frequency questionnaire to calculate MDS, healthy PDI (hPDI), and unhealthy PDI (uPDI). Multivariable logistic regression was used to determine associations.

**Results:**

Higher adherence to MDS (OR: 0.37; 95% CI: 0.18–0.73) and hPDI (OR: 0.41; 95% CI: 0.22–0.79) was associated with lower odds of GDM. Conversely, higher uPDI adherence was associated with increased odds of GDM (OR: 4.98; 95% CI: 2.39–9.34).

**Discussion:**

A Mediterranean diet or a healthy plant‐based diet may reduce GDM risk, while an unhealthy plant‐based diet may increase it, highlighting the importance of diet quality in GDM prevention.

## Graphical abstract

Higher adherence to Mediterranean (OR = 0.37) and healthy plant‐based diets (OR = 0.41) was associated with lower odds of gestational diabetes mellitus (GDM). In contrast, an unhealthy plant‐based diet (OR = 4.98) increased GDM risk among 276 Iranian pregnant women, highlighting the role of diet quality in GDM prevention.

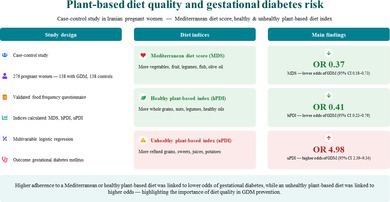

## 1 Introduction

Gestational Diabetes Mellitus (GDM) is defined as glucose intolerance that is first identified or onsets during pregnancy [1, 2]. As one of the most prevalent metabolic disorders of pregnancy, its global incidence is rising, with an estimated prevalence of 10.13% [3, 4]. Notably, Asian populations are at a disproportionately higher risk of developing GDM compared to their Western counterparts. In Iran, the estimated prevalence varies widely from 1.3% to 18.6%, affecting approximately 6% of all pregnancies [4]. This condition is a significant clinical concern as it predisposes both the mother and the child to a spectrum of adverse short‐ and long‐term health outcomes. For the mother, these include an elevated risk of preterm delivery, caesarean section, hypertensive disorders, and a heightened susceptibility to future type 2 diabetes, cardiovascular disease, obesity, and metabolic syndrome [1, 5, 6]. Offspring of mothers with GDM face increased risks of complications such as fetal hyperinsulinemia, neonatal hypoglycemia, jaundice, macrosomia, respiratory distress syndrome, and a lifelong predisposition to obesity and type 2 diabetes [1, 2, 5, 7]. Given this substantial health burden, identifying modifiable risk factors for the primary prevention of GDM is a critical public health priority.

Established non‐modifiable risk factors for GDM include advanced maternal age, family history of diabetes, and genetic predisposition. However, modifiable factors such as pre‐pregnancy overweight and obesity, excessive gestational weight gain, and an unhealthy lifestyle are increasingly recognised as pivotal targets for intervention [1, 8]. Among these, maternal diet before and during pregnancy has emerged as a key modifiable element that can significantly influence metabolic health and the risk of developing GDM [1, 8].

Dietary patterns provide a more holistic and comprehensive representation of habitual food consumption and its synergistic effects on health than the isolated examination of individual nutrients or foods [5]. Research has consistently highlighted the role of specific dietary patterns in either mitigating or exacerbating the risk of metabolic complications during pregnancy [2]. For instance, a Western dietary pattern, characterised by high intakes of red and processed meats, refined grains, and fast food, has been strongly associated with an increased risk of GDM [5]. Conversely, the Mediterranean Diet, which is rich in fruits, vegetables, legumes, whole grains, fish, and unsaturated fats from sources like olive oil, and low in red meat and processed foods, has been linked to a reduced risk of GDM in several studies [9]. More recently, the Plant‐Based Dietary Index (PDI) has gained attention as a tool for quantifying adherence to diets that emphasise plant‐based foods. This index distinguishes between healthy plant‐based foods (hPDI: Whole grains, fruits, vegetables, nuts, legumes, and oils) and less healthy plant‐based foods (uPDI: Fruit juices, refined grains, sweets, and sugar‐sweetened beverages), while also considering animal product intake [8]. Evidence suggests that higher adherence to overall and healthy plant‐based diets may be protective against conditions like type 2 diabetes [5, 10], cardiovascular disease [11], and potentially GDM [12]. In Iranian populations, plant‐based diet indices have been associated with metabolic health outcomes, including metabolic syndrome and biomarkers of insulin and inflammation [13, 14]. In particular, adherence to an HPDI has been linked to improved insulin‐related biomarkers, while an uPDI has been associated with higher insulin concentrations and inflammatory markers in Iranian older adults [14]. Additionally, major dietary patterns, including healthy and unhealthy patterns—have been shown to predict cardiovascular disease risk in Iranian adults [15], further supporting the relevance of diet quality for metabolic health in this population.

Despite the growing body of international evidence, the specific associations between the Mediterranean Diet Score (MDS) and the various facets of PDI (total, healthy, and unhealthy) and the odds of GDM remain underexplored in the Iranian population. Therefore, this case–control study aims to bridge this knowledge gap by assessing the association between adherence to the MDS and PDI indices and the odds of developing GDM among Iranian women.

## 2 Methods

### 2.1 Study Design and Participants

The current case–control study was conducted among pregnant women in Abadan, a city in Khuzestan province in southwestern Iran, who attended health centres between August 2022 and December 2022. A total of 138 pregnant women with GDM and 138 women without GDM, between the ages of 19 and 40 years, were selected by random sampling from local health centres. Women with a singleton pregnancy, no history of diabetes or GDM, and no medical conditions or medication use were eligible to participate in the study. Cases and controls were age‐matched 1:1 (±5 years). We excluded from the present analysis seven women in the control group and nine women in the case group who reported an energy intake above 4,000 kcal or did not complete the study. Following these exclusions, 138 cases and 136 controls remained for analysis.

### 2.2 GDM Assessment

Pregnant women who had not previously been diagnosed with diabetes were screened for GDM at 24–28 weeks of gestation with a 75‐g oral glucose tolerance test (OGTT). Plasma glucose levels were measured in the fasting state and at 1 and 2 h post‐glucose ingestion. Women were diagnosed with GDM if their fasting plasma glucose concentration was > 92 mg/dL and/or the 1‐ and 2‐h post‐challenge plasma glucose concentrations were ≥ 180 and > 153 mg/dL, respectively.

### 2.3 Dietary Intake Assessment

The usual dietary intake of the study participants was assessed using a validated 147‐item semiquantitative food frequency questionnaire (FFQ) administered by an interviewer [16, 17]. All women were asked to report their frequency of consumption of the foods listed in the FFQ over the past year, based on commonly used units or portion sizes. The FFQ consisted of a list of foods commonly consumed in the past year, which participants were asked to report in face‐to‐face interviews by trained dietitians on a daily, weekly, monthly, and yearly basis. The frequency with which each item was consumed was reported using standard household measuring equipment, and the measurements were converted to grams. Dietary intake was analysed using the Nutritionist IV software version 3.5 (N Squared Computing, San Bruno, CA, USA).

### 2.4 Mediterranean Diet Score Calculation

We calculated the MDS based on nine components [fruits and nuts, vegetables, fish, whole grains, legumes, and the ratio of monounsaturated fatty acids (MUFA) to saturated fatty acids (SFA), meats (red meat, poultry, and processed meats), dairy products, and wine] using the methodology of Trichopoulou et al. [18]. Women were categorised into tertiles based on their food group intake. The highest tertile of consumption was assigned a value of 2, the middle tertile a value of 1, and the lowest tertile a value of 0 for foods typical of the Mediterranean diet (fruits and nuts, fish, vegetables, legumes, whole grains, and the MUFA‐to‐SFA ratio). Conversely, a value of 2 was assigned to the highest consumption category, 1 to the medium category, and 0 to the lowest category for food groups not typical of the Mediterranean diet (meat and dairy products). Data on wine consumption were not included in this study. The final score was obtained by summing these values. It varies from 0 (low adherence) to 18 (high adherence).

### 2.5 Plant‐Based Dietary Index Calculation

To calculate the plant‐based dietary pattern, the method of Satija et al. was used [19]. This score is made up of three indices: The total PDI, the healthy PDI, and the unhealthy PDI. Foods were categorised into 18 groups based on nutrient similarity, including animal foods, healthy plant foods, and unhealthy plant foods. Healthy PDI included whole grains, fruits, vegetables, nuts, legumes, vegetable oils, and tea/coffee. Unhealthy PDI included fruit juices, sugar‐sweetened beverages, refined grains, potatoes, and sweets/desserts. Animal fat, dairy products, eggs, fish/seafood, meat, and miscellaneous animal products were also considered animal products. These foods were then converted into consumption quintiles, and each item was scored on a scale of 1 to 5. For the PDI, women in the highest and lowest quintiles of plant food consumption were given scores of 5 and 1, respectively. In addition, scores of 1 and 5 were assigned to the women in the highest and lowest quintiles of animal food consumption, respectively. For calculating the healthy PDI, participants with the highest and lowest intakes of healthy plant foods were assigned scores of 5 and 1, respectively. A score of 1 was also given for the highest consumption and 5 for the lowest consumption of unhealthy plant‐based and animal‐based foods. For the calculation of the unhealthy PDI, a score from 5 to 1 was assigned based on the consumption of unhealthy plant foods, ranked from highest to lowest. A score between 1 and 5 was also assigned to women based on their intake of animal and healthy plant foods, from highest to lowest. The scores were summed to provide each total PDI, healthy PDI, and unhealthy PDI index a score ranging from 18 to 90. Higher total scores for each index indicated higher adherence to the dietary pattern.

### 2.6 Assessment of Other Variables

Required information on other variables, including age, medical history, level of education, occupational status, living arrangements, occupational situation, and physical activity, was obtained from a structured questionnaire by face‐to‐face interview. Physical activity was evaluated using the International Physical Activity Questionnaire (IPAQ). The data are reported as mean ± SD for metabolic equivalent minutes per week (MET‐min/week) [20]. Pre‐pregnancy weight and height were obtained from the health centre's medical records. For weight gain during pregnancy, measure weight to the nearest 100 g using a balanced digital scale, dress lightly, and stand barefoot. Height was measured with a tape measure while the women were standing without shoes. Weight in kilograms was divided by height in meters squared to calculate body mass index (BMI). Regarding living facilities, women were asked which of 9 items their household owned: A car, a house, a washing machine, a dishwasher, an LCD or LED TV, an outdoor refrigerator, a computer or laptop, a handmade carpet, and an electric oven. Poor economic status was reported if participants had three or fewer of these items; medium economic status was considered for women with a score of 4–6; and high economic status was considered for those with a score of 7–9 [21].

### 2.7 Statistical Analysis

Following the recommendations of Willet et al. [22], food intake was adjusted for total energy intake using the residuals method for cases and controls. Histograms and skewness were used to analyse the normality of the data. Continuous variables that were normally distributed were summarised as means and standard deviations (SDs), and those that were not normally distributed were expressed as medians and interquartile ranges. Frequencies and percentages were used for categorical variables. An independent samples *t*‐test was used to compare continuous variables. Categorical variables were cross‐tabulated, and the chi‐squared test or Fisher's exact test was used to determine differences. Based on the MDS and the PDI, the study populations were divided into tertiles. The tertiles of the two scores were used to present the demographic data. Demographic data were analysed based on tertiles of the two scores using a one‐way analysis of variance. The chi‐squared test was used for both continuous and categorical variables. A multivariable logistic regression model was used to assess the association between MDS tertiles and PDI and to estimate the odds of GDM, with odds ratios (ORs) and 95% confidence intervals (CIs) calculated. Pre‐pregnancy BMI, physical activity, smoking status, and life facilities were included as potential confounders in Model 1. Potential confounders for model 2 were model 1 plus a family history of diabetes, eclampsia, hypertension, and abortion. For the trend across tertiles of two scores, *p*‐values were calculated. *p*‐values ≤ 0.05 were considered statistically significant.

## 3 Results

Table 1 shows the baseline characteristics of the study participants. Cases were more likely to have pre‐pregnancy weight (*p* < 0.001) and BMI (*p* < 0.001), low‐life facilities (*p* = 0.009), smoking (*p* = 0.03), family history of diabetes (*p* = 0.01), history of hypertension (*p* = 0.01), eclampsia (*p* = 0.03), and abortion (*p* = 0.03), also showed significant differences between groups. MDS (*p* = 0.01) and healthy PDI (*p* = 0.001) scores were significantly higher in the control group. Conversely, the cases had a substantially higher unhealthy PDI score than the controls (*p* < 0001).

**TABLE 1:** Study population demographic data among the GDM patients and healthy participants.

	GDM (*n* = 138)	Healthy (*n* = 138)	*p* *
**Demographic data**			
Age (year)	30.28 ± 5.01	29.07 ± 5.57	0.06
Pre‐pregnancy weight (kg)	73.48 ± 14.73	66.65 ± 12.95	**< 0.001**
Pre‐pregnancy BMI (kg/m^2^)	29.03 ± 6.14	26.39 ± 5.89	**< 0.001**
Pregnancy weight gain (kg)	6.91 ± 4.32	7.98 ± 6.44	0.10
Gestation length (week)	30.34 ± 4.09	31.03 ± 4.36	0.18
Education (*n*, %)			0.69
Illiterate or primary school	50 (47.2%)	56 (52.8%)	
High school and a diploma	51 (52.0%)	47 (48.0%)	
College and university	37 (52.9%)	33 (47.1%)	
Number of pregnancies (*n*, %)			0.27
First	32 (41.6%)	45 (58.4%)	
Second	41 (50.6%)	40 (49.4%)	
Third and more	64 (56.1%)	50 (43.9%)	
Gender of neonate (*n*, %)			0.47
Male	79 (52.3%)	72 (47.7%)	
Female	59 (48.0%)	64 (52.0%)	
Job (*n*, %)			0.72
Housewife	127 (50.0%)	127 (50.0%)	
Employee	4 (66.7%)	2 (33.3%)	
Self‐employed	7 (50.0%)	7 (50.0%)	
Life facilities			**0.009**
Low	16 (11.6%)	11 (8.1%)	
Medium	80 (58.0%)	59 (43.4%)	
High	42 (30.4%)	66 (48.5%)	
Active smoker (*n*, %)	9 (81.1%)	2 (18.2%)	**0.03**
Physical activity (MET/min/week)^a^	33.091 ± 3.37	33.52 ± 2.53	0.28
Family history of diabetes (*n*, %)	79 (58.1%)	57 (41.9%)	**0.01**
Family history of GDM (*n*, %)	19 (65.5%)	10 (34.5%)	0.08
Eclampsia (*n*, %)	7 (87.5%)	1 (12.5%)	**0.03**
History of hypertension (*n*, %)	19 (76.0%)	6 (24.0%)	**0.01**
Macrosomia history (*n*, %)	14 (60.9%)	9 (39.1%)	0.29
History of Caesarean section (*n*, %)	38 (56.7%)	29 (43.3%)	0.49
History of IUGF (*n*, %)	2 (50%)	2 (50%)	0.99
History of abortion (*n*, %)	45 (60.0%)	30 (40.0%)	**0.03**
History of stillbirth (*n*, %)	8 (72.7%)	3 (27.3%)	0.11
Multivitamin supplement use (*n*, %)	115 (48.9%)	120 (51.1%)	0.24
Folic acid supplement use (*n*, %)	135 (50.9%)	130 (49.1%)	0.30
Iron supplement use (*n*, %)	135 (50.9%)	130 (49.1%)	0.24
**Dietary intakes**			
Energy intake (kcal/d)	2392.99 ± 714.42	2308.35 ± 670.29	0.31
Carbohydrate (g/d)	328.01 ± 97.89	323.74 ± 94.78	0.71
Protein (g/d)	77.28 ± 22.53	75.89 ± 23.04	0.61
Total fat (g/d)	88.84 ± 33.81	82.50 ± 30.54	0.10
Fibre (g/d)	58.75 ± 23.55	58.43 ± 22.52	0.91
**Dietary scores**			
Med diet	7.78 ± 2.36	8.22 ± 2.32	**0.01**
Total PDI	53.79 ± 6.56	54.63 ± 6.76	0.29
Healthy PDI	52.92 ± 7.69	55.75 ± 6.62	**0.001**
Unhealthy PDI	56.56 ± 6.79	53.48 ± 6.65	**< 0.001**

*Note:* Data are presented as mean ± SD or per cent (*n*).

Abbreviations: BMI, body mass index; GDM, gestational diabetes mellitus; Med diet, Mediterranean diet; PDI, plant‐based diet index.

* Obtained from the independent‐sample *t*‐test, chi‐squared, or Fisher test, where appropriate.

^a^ Physical activity based on MET (metabolic equivalent min/week).

Table 2 presents participant characteristics by dietary score tertiles. Women in the highest tertile of healthy PDI were older (*p* < 0.05), whereas women in the highest tertile of unhealthy PDI were younger (*p* < 0.05) than women in the lowest tertile of healthy PDI. In addition, women in the highest tertile of unhealthy PDI had lower physical activity (*p* < 0.05) than women in the lowest tertile of unhealthy PDI.

**TABLE 2:** Study population characteristics according to tertiles of mediterranean diet and plant‐based diet index.

	Med diet		Total PDI		Healthy PDI		Unhealthy PDI	
	T1	T3	T1	T3	T1	T3	T1	T3
**Demographic data**								
Age (year)	29.72 ± 5.02	30.19 ± 5.66	29.79 ± 5.08	29.50 ± 5.65	28.01 ± 5.80	31.23 ± 4.85^a^	30.74 ± 4.98	28.22 ± 5.73^a^
Pre‐pregnancy weight (kg)	68.58 ± 14.28	71.10 ± 13.20	70.50 ± 13.06	68.61 ± 1516	68.12 ± 13.13	71.71 ± 15.02	71.21 ± 14.59	66.92 ± 12.84^a^
Pre‐pregnancy BMI (kg/m^2^)	27.42 ± 6.62	27.73 ± 5.21	28.15 ± 5.83	26.94 ± 7.14	27.23 ± 6.54	28.48 ± 6.78	27.72 ± 5.21	27.26 ± 6.89
Pregnancy weight gain (kg)	7.15 ± 4.09	7.21 ± 4.35	6.44 ± 3,86	8.15 ± 7.56^a^	7.51 ± 3.99	7.09 ± 3.89	7.57 ± 4.26	7.49 ± 7.79
Gestation length (week)	30.60 ± 3.79	30.87 ± 5.10	30.54 ± 4.93	30.48 ± 3.78	30.62 ± 5.11	31.07 ± 3.72	30.68 ± 4.71	30.62 ± 3.90
Education (*n*, %)								
Illiterate or primary school	35 (42.2%)	32 (40.5%)	40 (40.4%)	35 (37.6%)	39 (39.8%)	30 (34.1%)	35 (35.4%)	45 (53.6%)
High school and a diploma	28 (33.7%)	29 (36.7%)	32 (32.3%)	36 (38.7%)	38 (38.8%)	28 (31.8%)	36 (36.4%)	27 (32.1%)
College and university	20 (24.1%)	18 (22.8%)	27 (27.3%)	22 (23.7%)	21 (21.4%)	30 (34.1%)	28 (28.3%)	12 (14.3%)
Number of pregnancies (*n*, %)								
First	23 (27.7%)	24 (30.4%)	23 (23.2%)	25 (26.9%)	32 (32.7%)	25 (28.4%)	24 (24.2%)	26 (31.0%)
Second	22 (26.5%)	18 (22.8%)	35 (35.4%)	25 (26.9%)	28 (28.6%)	28 (31.8%)	27 (27.3%)	26 (31.0%)
Third and more	38 (45.8%)	36 (45.6%)	41 (41.4%)	42 (45.2%)	38 (38.8%)	25 (39.8%)	46 (46.5%)	32 (38.1%)
Gender of neonate (*n*, %)								
Male	47 (56.6%)	51 (64.6%)	52 (52.5%)	56 (60.2%)	52 (53.1%)	50 (56.8%)	58 (58.6%)	45 (53.6%)
Female	36 (43.4%)	28 (35.4%)	47 (47.5%)	56 (60.2%)	46 (46.9%)	38 (43.2%)	41 (41.4%)	39 (46.4%)
Job (*n*, %)								
Housewife	79 (95.2%)	75 (94.9%)	94 (94.9%)	87 (93.5%)	92 (93.9%)	79 (89.8%)	90 (90.9%)	82 (97.6%)
Employee	1 (1.2%)	1 (1.3%)	1 (1.0%)	3 (3.2%)	2 (2.0%)	3 (3.4%)	3 (3.0%)	0 (0.0%)
Self‐employed	3 (3.6%)	3 (3.8)	4 (4.0%)	3 (3.2%)	4 (4.1%)	6 (6.8%)	6 (6.1%)	2 (2.4%)
Life facilities								
Low	33 (39.8%)	36 (45.6%)	42 (42.4%)	35 (37.6%)	41 (41.8%)	33 (37.5%)	36 (36.4%)	46 (54.8%)
Medium	42 (50.6%)	40 (50.6%)	49 (49.5%)	48 (51.6%)	45 (45.9%)	45 (51.1%)	50 (50.5%)	36 (42.9%)
High	8 (9.6%)	3 (3.8%)	8 (8.1%)	10 (10.8%)	12 (12.2%)	10 (11.4%)	13 (13.1%)	2 (2.4%)
Active smoker (*n*, %)	4 (4.8%)	3 (3.8%)	1 (1.0%)	6 (6.5%)	5 (5.1%)	2 (2.3%)	3 (3.0%)	4 (4.8%)
Physical activity (MET/min/week) ^#^	33.21 ± 2.82	33.84 ± 2.91	33.49 ± 2.91	34.03 ± 2.84	33.37 ± 3.05	34.08 ± 2.87	34.26 ± 3.25	33.17 ± 2.40^a^
Family history of diabetes (*n*, %)	41 (49.4%)	32 (40.5%)	51 (51.5%)	45 (48.4%)	47 (48.0%)	43 (48.9%)	54 (54.5%)	35 (41.7%)
Family history of GDM (*n*, %)	8 (9.6%)	8 (10.1%)	6 (6.1%)	14 (15.1%)	13 (13.3%)	8 (9.1%)	8 (8.1%)	10 (11.9%)
Eclampsia (*n*, %)	1 (1.2%)	4 (5.1%)	0 (0.0%)	7 (7.5%)	1 (1.0%)	3 (3.4%)	3 (3.0%)	2 (2.4%)
History of hypertension (*n*, %)	6 (7.2%)	11 (13.9%)	4 (4.0%)	14 (15.1%)	7 (7.1%)	9 (10.2%)	9 (9.1%)	9 (10.7%)
Macrosomia history (*n*, %)	7 (8.4%)	6 (7.6%)	10 (10.1%)	10 (10.8%)	6 (6.1%)	5 (5.7%)	9 (9.1%)	5 (6.0%)
History of Caesarean section (*n*, %)	22 (26.5%)	18 (22.8%)	24 (24.2%)	20 (21.5%)	23 (23.5%)	19 (21.6%)	27 (27.3%)	16 (19.0%)
History of IUGF (*n*, %)	3 (3.6%)	1 (1.3%)	1 (1.0%)	2 (2.2%)	1 (1.0%)	0 (0.0%)	0 (0.0%)	1 (1.2%)
History of abortion (*n*, %)	24 (28.9%)	21 (26.6%)	23 (23.2%)	31 (33.3%)	23 (23.5%)	19 (21.6%)	32 (32.3%)	21 (25%)
History of stillbirth (*n*, %)	4 (4.8%)	5 (6.3%)	2 (2.0%)	6 (6.5%)	4 (4.1%)	2 (2.3%)	3 (3.0%)	2 (2.4%)
Multivitamin supplement use (*n*, %)	73 (88.0%)	64 (81.0%)	86 (86.9%)	80 (86.0%)	88 (89.8%)	75 (85.2%)	83 (83.8%)	76 (90.5%)
Folic acid supplement use (*n*, %)	79 (95.2%)	78 (98.7%)	95 (96.0%)	91 (97.8%)	95 (96.9%)	85 (96.6%)	95 (96.0%)	79 (94.0%)
Iron supplement use (*n*, %)	81 (97.6%)	77 (97.5%)	97 (98.0%)	86 (92.5%)	95 (96.9%)	85 (96.6%)	95 (96.0%)	80 (95.2%)

*Note:* Data are presented as mean ± SD.

Abbreviations: BMI, body mass index; GDM, gestational diabetes mellitus; Med diet, Mediterranean diet; PDI, plant‐based diet index.

^a^ Obtained from the independent‐sample *t*‐test.

Table 3 shows the crude and multivariable‐adjusted ORs and 95% CIs for GDM by tertiles of MDS, total PDI, healthy PDI, and unhealthy PDI scores. The odds of GDM were reduced across tertiles of the MDS (OR: 0.45; 95% CI: 0.24–0.84, Ptrend = 0.01) and healthy PDI (OR: 0.53; 95% CI: 0.29–0.94, Ptrend = 0.03) in the crude model. In contrast, higher adherence to the unhealthy PDI was associated with significantly higher odds of GDM in the highest tertile compared with the lowest tertile (OR: 2.56; 95% CI: 1.41–4.66; Ptrend = 0.002) in the crude model. In multivariable model 1, participants in the highest tertiles of the MDS (OR: 0.41; 95% CI: 0.21–0.80, Ptrend = 0.009) and healthy PDI (OR: 0.43; 95% CI: 0.23–0.81, Ptrend = 0.01) had lower odds of GDM compared with the lowest tertiles of scores. In model 1, higher unhealthy PDI adherence was associated with significantly higher odds of GDM in the highest tertile (OR: 4.20; 95% CI: 2.09–8.44, Ptrend < 0.001). In model 2, there was a significantly negative association between the highest tertile of MDS (OR: 0.37; 95% CI: 0.18–0.73, Ptrend = 0.006) and healthy PDI (OR: 0.41; 95% CI: 0.22–0.79, Ptrend = 0.008) and the odds of GDM compared with the lowest tertile. In contrast, there was a significant positive association between the highest tertile of unhealthy PDI and the odds of GDM (OR: 4.98; 95% CI: 2.39–9.34; Ptrend < 0.001) in model 2. However, there was no significant association between the highest tertile of total PDI and GDM, compared with the lowest tertile, in both crude and multivariable‐adjusted models.

**TABLE 3:** The association between the mediterranean diet and plant‐based diet index with odds of GDM.

Dietary scores	OR (95% CI) of GDM			*p*‐trend*
	T1	T2	T3	
**Med diet**				
Range of Med diet	< 7	7–9	≥ 9	
Case/Total	51/83	54/112	33/79	
Crude model	1 (Ref)	0.58 (0.32–1.04)	0.45 (0.24–0.84)	**0.01**
Model 1^a^	1 (Ref)	0.51 (0.27–0.94)	0.41 (0.21–80)	**0.009**
Model 2^b^	1 (Ref)	0.49 (0.26–0.92)	0.37 (0.18–0.73)	**0.006**
**Total PDI**				
Range of Total PDI	< 51	51–57	≥ 57	
Case/Total	51/99	45/82	42/93	
Crude model	1 (Ref)	1.14 (0.63–2.05)	0.77 (0.44–1.37)	0.39
Model 1^a^	1 (Ref)	1.06 (0.57–1.96)	0.74 (0.40–1.36)	0.34
Model 2^b^	1 (Ref)	1.02 (0.55–1.90)	0.63 (0.33–1.18)	0.19
**Healthy PDI**				
Case/Total	59/98	40/88	39/88	
Crude model	1 (Ref)	0.55 (0.31–0.99)	0.53 (0.29–0.94)	**0.03**
Model 1^a^	1 (Ref)	0.47 (0.25–0.88)	0.43 (0.23–0.81)	**0.01**
Model 2^b^	1 (Ref)	0.42 (0.22–0.81)	0.41 (0.22–0.79)	**0.008**
**Unhealthy PDI**				
Case/Total	64/99	54/91	49/84	
Crude model	1 (Ref)	2.67 (1.48–4.80)	2.56 (1.41–4.66)	**0.002**
Model 1^a^	1 (Ref)	2.79 (1.48–5.28)	4.20 (2.09–8.44)	**< 0.001**
Model 2^b^	1 (Ref)	3.24 (1.68–6.25)	4.98 (2.39–9.34)	**< 0.001**

Abbreviations: GDM: gestational diabetes mellitus; Med diet: Mediterranean diet; PDI: plant‐based diet index.

* The logistic regression model, with the lowest tertile as a reference.

^a^ Model 1: Adjusted for pre‐pregnancy BMI, physical activity, smoking status, and life facilities.

^b^ Model 2: Adjusted for model 1 + family history of diabetes, eclampsia, hypertension, and abortion.

## 4 Discussion

This case–control study of 276 Iranian pregnant women provides significant evidence on the association between dietary patterns and the odds of GDM. Our key finding is that greater adherence to the MDS and the hPDI is associated with a substantially lower odds of GDM, both before and after adjusting for potential confounders. Conversely, a higher uPDI score is strongly associated with an increased risk of GDM. No significant association is observed for the overall PDI.

Our results regarding the protective role of the Mediterranean diet align consistently with a growing body of international literature. The inverse relationship we observed between MDS and GDM odds is supported by studies linking this dietary pattern to improved glycemic control during pregnancy. For instance, Karamanos et al. reported that higher adherence to the MDS was inversely correlated with fasting, 1‐h, and 2‐h plasma glucose levels following a 75 g OGTT, suggesting a direct beneficial effect on glucose metabolism [23]. Meta‐analyses and extensive prospective studies further corroborate this protective effect. A systematic review by Jafari Nasab et al. concluded that adherence to the Mediterranean diet was associated with a reduced risk of GDM [9]. Furthermore, two extensive cohort studies found that women with the highest adherence to the MDS had a 24% [24] and 41% [25] lower risk of developing GDM, respectively. Furthermore, as highlighted by Tranidou et al. [26], the low meat intake characteristic of this diet may be a particularly important factor in reducing the risk of GDM.

Regarding plant‐based diets, our findings add to a complex but generally supportive narrative. The significant reduction in GDM odds associated with the hPDI, which emphasises whole grains, fruits, vegetables, nuts, legumes, and healthy oils, is consistent with several previous studies. A cohort study by Wang et al. [27]. conducted in China demonstrated that a higher overall PDI during pregnancy was significantly associated with a reduced risk of GDM. Similarly, a prospective birth cohort study by Bazshahi et al. [28]. found that higher adherence to both PDI and hPDI in early pregnancy was linked to a significantly lower risk of GDM. Most notably, an extensive prospective cohort study from the Nurses' Health Study II by Chen et al. [29]. found that greater pre‐pregnancy adherence to PDI and hPDI was associated with a 30% and 25% lower risk of GDM, respectively. The beneficial effects of a healthy plant‐based diet are attributed to its high fibre content, which slows glucose absorption and improves insulin sensitivity; antioxidants that combat oxidative stress; and micronutrients like magnesium, which play a role in glucose metabolism [27, 28].

Our findings are further supported by previous research conducted in Iranian populations. Amini et al. [13]. investigated the association between plant‐based diet indices and metabolic syndrome among 178 older Iranian adults. They found that although individual components, such as HDL‐cholesterol, differed across tertiles of hPDI and uPDI, there was no significant association between any of the plant‐based diet indices and metabolic syndrome after adjustment for confounders. Although our study focused on GDM rather than metabolic syndrome, the parallel findings, specifically, the significant associations observed for hPDI and uPDI with metabolic parameters (HDL‐cholesterol in their study; GDM odds in our study) but not for overall PDI, suggest that distinguishing between healthy and unhealthy plant foods is crucial when assessing metabolic outcomes. Similarly, Shahinfar et al. [14]. examined the association between plant‐based diet indices and inflammatory and insulin biomarkers in older Iranian adults. Their findings demonstrated that greater adherence to PDI and hPDI was associated with lower serum insulin concentrations and improved insulin sensitivity, whereas greater adherence to uPDI was associated with higher insulin levels. These results align closely with our observation that hPDI reduces GDM odds (OR: 0.41). At the same time, uPDI increases GDM risk (OR: 4.98), reinforcing the biological plausibility that dietary influences on insulin and inflammation mediate the relationship between plant‐based diets and GDM. Furthermore, Shahavandi et al. [15]. reported that a healthy dietary pattern score was negatively associated with systolic blood pressure and Framingham Risk Score in Iranian adults. In contrast, an unhealthy pattern was associated with lower HDL‐cholesterol. Collectively, these studies underscore that the quality, not merely the quantity, of plant‐based food consumption determines metabolic health outcomes across different life stages and conditions.

Crucially, our study underscores the importance of diet quality by clearly distinguishing between healthy and unhealthy plant‐based foods. The uPDI, which includes fruit juices, refined grains, sweets, and sugar‐sweetened beverages, was associated with a markedly higher risk of GDM. This suggests that merely avoiding animal products is insufficient for protection if the resulting diet is rich in processed carbohydrates and sugars. These foods are known to contribute to hyperglycemia and insulin resistance, thereby increasing the risk of GDM [27, 28].

The Mediterranean diet offers numerous health benefits due to its diverse components. This diet is rich in monounsaturated fatty acids (MUFAs), primarily due to the extensive use of olive oil in cooking. It also contains polyphenols, natural antioxidants,fibre, and low saturated fat. Adhering to this MDS may lower the risk of GDM, reduce systemic oxidative stress, and decrease circulating levels of inflammatory biomarkers [23, 30]. It is important to note that some studies, such as the one by He et al. [31]., found a protective association with plant‐based patterns, while others, such as Mak et al. [32]., found no significant association. These discrepancies may be explained by whether the studied pattern emphasised healthy vs. unhealthy plant foods, a distinction that our study and others, such as Chen et al. [29]., explicitly make.

A novel aspect of the present study is the simultaneous evaluation of the Mediterranean Diet Score alongside healthy and unhealthy plant‐based dietary indices in relation to GDM within the same population. While previous cohort studies and meta‐analyses have separately reported inverse associations for Mediterranean diets [9, 25] or healthy plant‐based diets [28, 29], few have directly compared their effect sizes or examined their mutual independence. Our study incrementally shows that both high MDS and high hPDI adherence are associated with similarly reduced odds of GDM (approximately 60% lower odds in fully adjusted models); the unhealthy plant‐based pattern (uPDI) is a strong risk factor (OR ~5), and these associations are independent of each other and of traditional risk factors. This comparative insight, emphasising diet quality over plant‐based status alone, extends prior international evidence to an Iranian population, where data on these combined dietary patterns have been scarce. Nonetheless, causal inference is limited by the case–control design, and our findings should be confirmed in prospective Iranian cohorts.

This study has several strengths, including the use of a validated and comprehensive FFQ administered by trained experts, which enhances the reliability of dietary assessment, and careful adjustment for a wide array of potential confounders. However, limitations must be acknowledged. The case–control design precludes inference of causality. Importantly, because dietary intake was assessed after GDM diagnosis, we cannot rule out reverse causation. Specifically, women diagnosed with GDM may have modified their eating habits following medical advice or dietarycounselling, which could have influenced the reported dietary patterns and potentially attenuated or altered the observed associations. This temporal ambiguity is an inherent limitation of retrospective dietary assessment in case–control studies. Additionally, dietary intake was self‐reported and subject to recall bias, and the complexity of the FFQ may lead to reporting errors. Furthermore, we did not account for nutritional behaviours such as meal timing and frequency. Therefore, our findings should be confirmed by extensive prospective cohort studies or randomised controlled trials that assess diet before the diagnosis of GDM.

## 5 Conclusion

In conclusion, our study demonstrates that the quality of dietary patterns is critically important in modulating the risk of GDM among Iranian women. Adherence to a Mediterranean diet or a healthy plant‐based diet rich in whole grains, fruits, and vegetables is associated with a lower likelihood of GDM. In contrast, a diet high in unhealthy plant‐based foods, such as refined grains and sweets, is associated with a higher risk. These findings highlight the importance of promoting high‐quality, plant‐centred dietary patterns for the primary prevention of GDM.

## Author Contributions

**Farnush Bakhshimoghaddam:** investigation, writing – original draft, writing – review and editing, methodology, software, project administration. **Masoud Veissi:** supervision. **Anahita Mansoori:** supervision. **Mina Bahrami:** writing – original draft. **Masoume Gharang:** methodology, data curation.

## Funding

This study was supported by the Student Research Committee of Ahvaz Jundishapur University of Medical Sciences, under grant 03s86, supported this research.

## Disclosure

The authors have nothing to report.

## Ethics Statement

The study was conducted in accordance with the Declaration of Helsinki and approved by the Ethics Committee of Ahvaz Jundishapur University of Medical Sciences (Ethics Code: IR.AJUMS.REC.1403.661). Informed written consent was obtained from all participants in the study.

## Consent

The authors have nothing to report.

## Conflicts of Interest

The authors declare no conflicts of interest.

## Data Availability

The data that support the findings of this study are available from the corresponding author upon reasonable request.

## References

[1] V. Lambert , S. E. Muñoz , C. Gil , and M. D. Román , “Maternal Dietary Components in the Development of Gestational Diabetes Mellitus: A Systematic Review of Observational Studies to Timely Promotion of Health,” Nutrition Journal 22, no. 1 (2023): 15. 36879315 10.1186/s12937-023-00846-9 PMC9990275

[2] H. O'Hara , J. Taylor , and J. V. Woodside , “The Association of Specific Dietary Patterns With Cardiometabolic Outcomes in Women With a History of Gestational Diabetes Mellitus: A Scoping Review,” Nutrients 15, no. 7 (2023): 1613. 10.3390/nu15071613 PMC10097232 37049454

[3] J. Hu , E. Oken , I. M. Aris , et al., “Dietary Patterns During Pregnancy Are Associated With the Risk of Gestational Diabetes Mellitus: Evidence From a Chinese Prospective Birth Cohort Study,” Nutrients 11, no. 2 (2019): 405. 30769927 10.3390/nu11020405 PMC6412704

[4] S. Nazarpour , M. Simbar , Z. Kiani , N. Khalaji , M. Khorrami Khargh , and Z. Naeiji , “The Relationship Between Quality of Life and Some Mental Problems in Women With Gestational Diabetes Mellitus (GDM): A Cross‐Sectional Study,” BMC Psychiatry 24, no. 1 (2024): 1–12. 39026253 10.1186/s12888-024-05960-4 PMC11256570

[5] Y. Zhu , Q. Zheng , L. Huang , et al., “The Effects of Plant‐Based Dietary Patterns on the Risk of Developing Gestational Diabetes Mellitus: A Systematic Review and Meta‐Analysis,” PLoS One 18, no. 10 (2023): e0291732. 37792722 10.1371/journal.pone.0291732 PMC10550137

[6] C. Zhang and Y. Ning , “Effect of Dietary and Lifestyle Factors on the Risk of Gestational Diabetes: Review of Epidemiologic Evidence,” American Journal of Clinical Nutrition 94, no. 6 Suppl (2011): 1975s–1979s. 21613563 10.3945/ajcn.110.001032 PMC3364079

[7] H. Wang , H. Jiang , L. Yang , and M. Zhang , “Impacts of Dietary Fat Changes on Pregnant Women With Gestational Diabetes Mellitus: A Randomized Controlled Study,” Asia Pacific Journal of Clinical Nutrition 24, no. 1 (2015): 58–64. 25740743 10.6133/apjcn.2015.24.1.19

[8] X. Q. Chen , Q. Zheng , Y. P. Liao , et al., “Association Between Plant‐Based or Animal‐Based Dietary Pattern and Plasma Glucose During Oral Glucose Tolerance Test Among Chinese Women With Gestational Diabetes Mellitus: A Prospective Cohort Study,” BMJ Open 13, no. 10 (2023): e075484. 10.1136/bmjopen-2023-075484 PMC10603417 37879688

[9] S. Jafari Nasab , M. Ghanavati , C. C. Clark , and M. Nasirian , “Adherence to Mediterranean Dietary Pattern and the Risk of Gestational Diabetes Mellitus: A Systematic Review and Meta‐Analysis of Observational Studies,” Nutrition & Diabetes 14, no. 1 (2024): 55. 39039056 10.1038/s41387-024-00313-2 PMC11263544

[10] F. Qian , G. Liu , F. B. Hu , S. N. Bhupathiraju , and Q. Sun , “Association Between Plant‐Based Dietary Patterns and Risk of Type 2 Diabetes: A Systematic Review and Meta‐Analysis,” JAMA Internal Medicine 179, no. 10 (2019): 1335–1344. 31329220 10.1001/jamainternmed.2019.2195 PMC6646993

[11] J. Quek , G. Lim , W. H. Lim , et al., “The Association of Plant‐Based Diet With Cardiovascular Disease and Mortality: A Meta‐Analysis and Systematic Review of Prospect Cohort Studies,” Frontiers in Cardiovascular Medicine 8 (2021): 756810. 34805312 10.3389/fcvm.2021.756810 PMC8604150

[12] B. Zamani , A. Milajerdi , H. Tehrani , N. Bellissimo , N. R. Brett , and L. Azadbakht , “Association of a Plant‐Based Dietary Pattern in Relation to Gestational Diabetes Mellitus,” Nutrition and Dietetics 76, no. 5 (2019): 589–596. 30680868 10.1111/1747-0080.12512

[13] M. R. Amini , H. Shahinfar , F. Djafari , et al., “The Association Between Plant‐Based Diet Indices and Metabolic Syndrome in Iranian Older Adults,” Nutrition and Health 27, no. 4 (2021): 435–444. (PMID: 33626298). 33626298 10.1177/0260106021992672

[14] H. Shahinfar , M. R. Amini , N. Payandeh , et al., “The Link Between Plant‐Based Diet Indices With Biochemical Markers of Bone Turn Over, Inflammation, and Insulin in Iranian Older Adults,” Food Science & Nutrition 9, no. 6 (2021): 3000–3014. 34136166 10.1002/fsn3.2258 PMC8194905

[15] M. Shahavandi , M. R. Amini , H. Shahinfar , and S. Shab‐Bidar , “Major Dietary Patterns and Predicted Cardiovascular Disease Risk in an Iranian Adult Population,” Nutrition and Health 27, no. 1 (2021): 27–37. 32867574 10.1177/0260106020952591

[16] F. H. Esfahani , G. Asghari , P. Mirmiran , and F. Azizi , “Reproducibility and Relative Validity of Food Group Intake in a Food Frequency Questionnaire Developed for the Tehran Lipid and Glucose Study,” Journal of Epidemiology 20, no. 2 (2010): 150–158. 20154450 10.2188/jea.JE20090083 PMC3900814

[17] P. Mirmiran , F. H. Esfahani , Y. Mehrabi , M. Hedayati , and F. Azizi , “Reliability and Relative Validity of an FFQ for Nutrients in the Tehran Lipid and Glucose Study,” Public Health Nutrition 13, no. 5 (2009): 654–662. 19807937 10.1017/S1368980009991698

[18] A. Trichopoulou , T. Costacou , C. Bamia , and D. Trichopoulos , “Adherence to a Mediterranean Diet and Survival in a Greek Population,” New England Journal of Medicine 348, no. 26 (2003): 2599–2608. 12826634 10.1056/NEJMoa025039

[19] A. Satija , S. N. Bhupathiraju , D. Spiegelman , et al., “Healthful and Unhealthful Plant‐Based Diets and the Risk of Coronary Heart Disease in US Adults,” Journal of the American College of Cardiology 70, no. 4 (2017): 411–422. 28728684 10.1016/j.jacc.2017.05.047 PMC5555375

[20] M. B. Moghaddam , F. B. Aghdam , M. A. Jafarabadi , H. Allahverdipour , S. D. Nikookheslat , and S. Safarpour , “The Iranian Version of International Physical Activity Questionnaire (IPAQ) in Iran: Content and Construct Validity, Factor Structure, Internal Consistency and Stability,” World Applied Sciences Journal 18, no. 8 (2012): 1073–1080.

[21] G. Mishra , K. Ball , J. Arbuckle , and D. Crawford , “Dietary Patterns of Australian Adults and Their Association With Socioeconomic Status: Results From the 1995 National Nutrition Survey,” European Journal of Clinical Nutrition 56, no. 7 (2002): 687–693. 12080411 10.1038/sj.ejcn.1601391

[22] W. C. Willett , G. R. Howe , and L. H. Kushi , “Adjustment for Total Energy Intake in Epidemiologic Studies,” American Journal of Clinical Nutrition 65, no. 4 (1997): 1220S–1228S. 9094926 10.1093/ajcn/65.4.1220S

[23] B. Karamanos , A. Thanopoulou , E. Anastasiou , et al., “Relation of the Mediterranean Diet With the Incidence of Gestational Diabetes,” European Journal of Clinical Nutrition 68, no. 1 (2014): 8–13. 24084515 10.1038/ejcn.2013.177

[24] D. K. Tobias , C. Zhang , J. Chavarro , et al., “Prepregnancy Adherence to Dietary Patterns and Lower Risk of Gestational Diabetes Mellitus,” American Journal of Clinical Nutrition 96, no. 2 (2012): 289–295. 22760563 10.3945/ajcn.111.028266 PMC3396443

[25] F. Mohtashaminia , F. Hosseini , A. Jayedi , et al., “Adherence to the Mediterranean Diet and Risk of Gestational Diabetes: A Prospective Cohort Study,” BMC Pregnancy and Childbirth 23, no. 1 (2023): 647. 37684573 10.1186/s12884-023-05960-4 PMC10486001

[26] A. Tranidou , T. Dagklis , E. Magriplis , et al., “Pre‐Pregnancy Adherence to Mediterranean Diet and Risk of Gestational Diabetes Mellitus: A Prospective Cohort Study in Greece,” Nutrients 15, no. 4 (2023): 848. 36839206 10.3390/nu15040848 PMC9967881

[27] H. Wang , L. Huang , L. Lin , et al., “The Overall Plant‐Based Diet Index During Pregnancy and Risk of Gestational Diabetes Mellitus: A Prospective Cohort Study in China,” British Journal of Nutrition 126, no. 10 (2021): 1519–1528. 33468274 10.1017/S0007114521000234

[28] E. Bazshahi , S. Pourreza , A. Jayedi , M. Mirmohammadkhani , A. Emadi , and S. Shab‐Bidar , “Adherence to Plant‐Based Diet During Pregnancy and Risk of Gestational Diabetes: A Prospective Birth Cohort Study,” BMC Nutrition 10, no. 1 (2024): 139. 39425217 10.1186/s40795-024-00949-4 PMC11488182

[29] Z. Chen , F. Qian , G. Liu , et al., “Prepregnancy Plant‐Based Diets and the Risk of Gestational Diabetes Mellitus: A Prospective Cohort Study of 14,926 Women,” American Journal of Clinical Nutrition 114, no. 6 (2021): 1997–2005. 34510175 10.1093/ajcn/nqab275 PMC8634573

[30] F. Mahjoub , H. Ben Jemaa , F. Ben Sabeh , N. Ben Amor , A. Gamoudi , and H. Jamoussi , “Impact of Nutrients and Mediterranean Diet on the Occurrence of Gestational Diabetes,” Libyan Journal of Medicine 16, no. 1 (2021): 1930346. 34024269 10.1080/19932820.2021.1930346 PMC8158182

[31] J.‐R. He , M.‐Y. Yuan , N.‐N. Chen , et al., “Maternal Dietary Patterns and Gestational Diabetes Mellitus: A Large Prospective Cohort Study in China,” British Journal of Nutrition 113, no. 8 (2015): 1292–1300. 25821944 10.1017/S0007114515000707

[32] J. K. Mak , N. M. Pham , A. H. Lee , et al., “Dietary Patterns During Pregnancy and Risk of Gestational Diabetes: A Prospective Cohort Study in Western China,” Nutrition Journal 17 (2018): 1–11. 30454043 10.1186/s12937-018-0413-3 PMC6245777

